# A Novel Secreted Cysteine-Rich Anionic (Sca) Protein from the Citrus Postharvest Pathogen *Penicillium digitatum* Enhances Virulence and Modulates the Activity of the Antifungal Protein B (AfpB)

**DOI:** 10.3390/jof6040203

**Published:** 2020-10-02

**Authors:** Sandra Garrigues, Jose F. Marcos, Paloma Manzanares, Mónica Gandía

**Affiliations:** Food Biotechnology Department, Instituto de Agroquímica y Tecnología de Alimentos (IATA), Consejo Superior de Investigaciones Científicas (CSIC), Paterna, 46980 Valencia, Spain; s.garrigues@wi.knaw.nl (S.G.); jmarcos@iata.csic.es (J.F.M.); pmanz@iata.csic.es (P.M.)

**Keywords:** antifungal protein (AFP), *Penicillium digitatum*, AfpB, postharvest decay, citrus fruit, virulence, cysteine-rich protein (CRP)

## Abstract

Antifungal proteins (AFPs) from ascomycete fungi could help the development of antimycotics. However, little is known about their biological role or functional interactions with other fungal biomolecules. We previously reported that AfpB from the postharvest pathogen *Penicillium digitatum* cannot be detected in the parental fungus yet is abundantly produced biotechnologically. While aiming to detect AfpB, we identified a conserved and novel small Secreted Cysteine-rich Anionic (Sca) protein, encoded by the gene PDIG_23520 from *P. digitatum* CECT 20796. The *sca* gene is expressed during culture and early during citrus fruit infection. Both null mutant (Δ*sca*) and Sca overproducer (Sca^op^) strains show no phenotypic differences from the wild type. Sca is not antimicrobial but potentiates *P. digitatum* growth when added in high amounts and enhances the in vitro antifungal activity of AfpB. The Sca^op^ strain shows increased incidence of infection in citrus fruit, similar to the addition of purified Sca to the wild-type inoculum. Sca compensates and overcomes the protective effect of AfpB and the antifungal protein PeAfpA from the apple pathogen *Penicillium expansum* in fruit inoculations. Our study shows that Sca is a novel protein that enhances the growth and virulence of its parental fungus and modulates the activity of AFPs.

## 1. Introduction

Infections caused by fungal pathogens pose a serious risk to human health, food production and security [1,2]. In agriculture, fungal plant pathogens are of great economic importance because they threaten the production of crops and can cause severe postharvest diseases, with an increasing incidence trend in the last decades [1,3]. To combat fungal infections, multiple chemical fungicide treatments are widely applied, which have negative effects on animal and human health and the environment. Currently, there are very few classes of fungicides available to treat (and prevent) fungal infections, leading to a rapid increase in resistance against the existing compounds [4]. Consequently, new antifungal strategies are urgently needed, and interest is focused in novel and sustainable antifungal agents with high efficacy, limited toxicity, low production costs and with different modes of action from the currently existing ones [5,6,7,8,9,10].

Antimicrobial peptides (AMPs) are a broad class of peptides and proteins with direct killing activity produced by organisms all along the phylogenetic scale [11], and serve as natural defenses against infections caused by microbial pathogens, mainly bacteria and fungi. AMPs can be classified as cationic or anionic, based on their net charge [12], and are gaining extensive attention as natural antibiotics among the scientific community.

Antifungal proteins (AFPs) of fungal origin are a specific class of AMPs that have been considered as promising alternatives to chemical fungicides [13]. AFPs are small, secreted, cationic, cysteine-rich proteins (CRPs) that fold into compact structures stabilized by disulphide bonds [14], which makes them highly resistant to heat, proteolysis and extreme pH [15,16], and exhibit antifungal activity at micromolar concentrations [16,17,18,19]. AFPs are encoded as pre-pro-proteins with a signal peptide (SP) at the N-termini involved in protein secretion and a pro-sequence whose function is still unclear [13,20]. The proteins PAF from *Penicillium chrysogenum* and AFP from *Aspergillus giganteus* are the most studied and characterized AFPs to date [20,21]. However, the number of experimentally characterized AFPs and predicted AFP-like sequences in filamentous fungi is continuously rising given the increasing availability of fungal genome sequences. Based on phylogenetic analysis, our previous studies showed that a given fungal genome encodes up to three distinct AFPs and grouped fungal AFPs into three different classes (A, B and C) [22]. This classification expanded the two clusters previously reported [23,24], although a more recent group of AFPs has been described [18], suggesting the existence of new unidentified classes of AFPs yet to be characterized.

*Penicillium digitatum* is the main citrus postharvest pathogen and causes green mold disease in citrus fruits, being responsible for important economic losses worldwide [25,26,27]. It only encodes one *afp* gene from class B, named *afpB* [22], and the corresponding protein AfpB could only be detected and produced when biotechnologically expressed in the yeast *Pichia pastoris*, the filamentous fungus *P. digitatum* [16] and in *Nicotiana benthamiana* plants [28]. AfpB shows potent in vitro inhibitory activity and was the first AFP protein for which self-inhibitory activity against its parental fungus was reported [16]. Additionally, AfpB has demonstrated high in vivo inhibition against *Botrytis cinerea* infection on tomato leaves [29], suggesting that AfpB could be a promising candidate as a bio-fungicide with no toxic effect on human red blood cells [16]. A detailed understanding of the mode of action is required for the potential application of AFPs as antifungal compounds. The most studied AFPs have similarities as well as differences in their mode of action against sensitive fungi, particularly in relation to whether they induce the cell wall integrity pathway, are internalized as part of their antifungal mechanism, induce disturbances in the intracellular Ca^+2^ concentration, induce the production of reactive oxygen species (ROS) or affect intracellular signaling (reviewed in [14]). In this context, we have initiated the study of the mode of action of AfpB from *P. digitatum*. From our previous work, we showed that this protein induces the phosphorylation of mitogen-activated protein kinases (MAPK) [30], establishing a connection between AfpB and cell wall stress. Additionally, we recently proposed that the AfpB killing activity occurs in three steps: (i) interaction with the cell wall; (ii) rapid cell internalization; (iii) ROS-mediated regulated cell death [31]. However, nothing is known about possible functional interactions between AFPs in general, and AfpB in particular, with other biomolecules produced by the parental fungi.

In this study, we have identified a novel small Secreted Cysteine-rich and Anionic (Sca) protein in *P. digitatum* that modulates the activity of AfpB towards the parental fungus. In order to characterize this novel protein, we determined its gene expression pattern, generated Sca-null (Δ*sca*) and -overproducer (Sca^op^) *P. digitatum* strains and tested the putative activity of the protein and its effect on AfpB antifungal activity. Our data reveal that the Sca-encoding gene is expressed very early during citrus fruit infection. Additionally, Sca shows neither antifungal nor antibacterial activity but rather enhances antifungal activity of AfpB in vitro and increases incidence of *P. digitatum* infection in vivo, overcoming the protective effect of AFPs during citrus fruit infection.

## 2. Materials and Methods

### 2.1. Microorganisms, Media and Culture Conditions

*P. digitatum* CECT 20796 (isolate PHI26) [25] and all transformant strains were cultured in potato dextrose agar (PDA; Difco, Sparks, MD, USA) for 7 days at 25 °C. Growth in solid PDA medium was analyzed by depositing 5 µL of conidial suspension (5 × 10^4^ conidia/mL) in the center of PDA plates and daily measurement of growth diameter. Conidial growth in liquid medium was assessed in 100 mL of potato dextrose broth (PDB; Difco) at 25 °C with shaking.

Different vectors used for fungal transformation were cloned and propagated in *Escherichia coli* JM109 cultured in Luria Bertani (LB) medium supplemented with the corresponding antibiotics (25 µg/mL chloramphenicol; 50 µg/mL kanamycin or 100 µg/mL spectinomycin) at 37 °C. *Agrobacterium tumefaciens* AGL-1 strain was cultured in LB medium with 20 µg/mL rifampicin at 28 °C. Yeast *Saccharomyces cerevisiae* BY4741 was incubated at 30 °C. For antibacterial assays, *E. coli* JM109 and *Bacillus subtilis* CECT 498 were grown in LB medium with shaking at 37 °C.

### 2.2. Protein Identification, Structure and Functional Domain Prediction

For Sca (PDIG_23520) identification, *P. digitatum* CECT 20796 cell-free supernatant was collected after 21 days of growth in PDB, centrifuged and tenfold concentrated (Speedvac, Concentrator plus, Eppendorf, Hamburg, Germany). Total proteins were separated by SDS-PAGE [32] using SDS-16% polyacrylamide gels calibrated with pre-stained protein size-standard SeeBlue^®^ (ThermoFischer Scientific, Waltham, MA, USA) and visualized by Coomassie blue staining. The ~12 kDa protein band was cut and analyzed in the proteomics facility of ‘Servei Central de Suport a la Investigació Experimental’ (SCSIE) of University of Valencia (Spain).

For protein identification, peptide mass fingerprinting (PMF) and N-terminal sequencing were performed (Supplementary Figure S1). For PMF, samples were subjected to trypsin digestion and the resulting mixtures were analyzed on a 5800 Matrix-assisted laser desorption/ionization (MALDI)-Time-of-flight (TOF)/TOF in positive reflectron mode (3000 shots at every position). Five of the most intense precursors (according to the threshold criteria: minimum signal-to-noise: 10, minimum cluster area: 500, maximum precursor gap: 200 parts per million (ppm), maximum fraction gap: 4) were selected for every position for the tandem mass spectrometry (MS/MS) analysis. MS/MS data were acquired using the default 1 kV MS/MS method. The MS and MS/MS information was sent to MASCOT via the Protein Pilot software (AB Sciex, Madrid, Spain). The N-terminal sequence of Sca was determined by the protein chemistry facility at the Margarita Salas Center for Biological Research (CIB-CSIC, Madrid, Spain) by N-terminal Edman degradation method [33].

Sequences from different Sca homologs among filamentous fungi were identified through BLASTP searches carried out at the National Center for Biotechnology Information server (https://blast.ncbi.nlm.nih.gov/Blast.cgi) (detailed in Supplementary Figure S2). A putative SP was predicted using the SignalP v4.0 server [34]. The theoretical molecular weight (MW) and isoelectric point (pI) of the mature Sca and Sca homologs were examined with the Compute pI/MW and ProtParam tools of the ExPASy Proteomics Server (https://www.expasy.org/). Sca putative Pfam domains were searched with the Pfam v33.1 online tool from the EMBL-EBI server [35] (http://pfam.xfam.org/).

The Sca secondary structure was predicted by the JPred4 server [36]. Accession numbers of protein sequences used in the alignments were obtained at the UniProt (http://www.uniprot.org) server. Amino acid sequence alignments were performed with the Clustal W algorithm [37] included in the MEGA v10 package [38], and alignments were further refined with minor adjustments.

### 2.3. Total RNA Isolation, Quantitative RT-PCR and Relative Expression

Time course experiments in solid and liquid media to collect mycelia for RNA isolation were performed as previously described [39].

Total RNA from (i) fungal conidia, (ii) time course experiments of *P. digitatum* CECT 20796 grown in PDB or PDA and (iii) infected fruits was isolated following previously described procedures [39,40]. Treatment with RNase-free DNase (ThermoFischer Scientific), synthesis of first-strand cDNA for quantitative RT-PCR assays, and determination of relative changes of gene expression between samples were conducted as described [39,41]. As independent housekeeping genes, *P. digitatum* β-tubulin [42], ribosomal protein L18a [43] and 18S rRNA [44] genes were simultaneously used (Supplementary Table S1).

### 2.4. Generation of Sca Null and Overproducer Strains

The AccuPrime High Fidelity polymerase (Invitrogen, Eugene, OR, USA) was used for all PCR procedures, and the resulting DNA products were sequenced for verification. All primer sequences and their location are shown in Supplementary Table S2 and Supplementary Figures S3 and S4. The genetic construct to disrupt the *sca* gene by homologous recombination was generated by fusion PCR [45]. Previously described procedures were applied to obtain the vector pGKO2_Δ*sca* [22,41]. Briefly, the hygromycin-resistant cassette (*hph*) used as positive selection marker was flanked by fungal DNA fragments of 1035 bp (primers OJM449 and OJM450) and 991 bp (primers OJM451 and OJM452) amplified from *P. digitatum* CECT 20796 genomic DNA. The fusion PCR fragment obtained was purified and ligated into pGEM-T Easy Vector System I (Promega, Madison, WI, USA). *Spe*I and *Hin*dIII restriction sites were used to insert the construct into the binary vector pGKO2 [46], whose T-DNA also contains the Herpes simplex virus-1 thymidine kinase gene (*HSVtk*) used as negative selection marker, to obtain the plasmid pGKO2_Δ*sca*.

In parallel, to generate specific gene constructs for Sca overproduction, the FungalBraid (FB) modular cloning approach was applied (Supplementary Figure S4) [47,48]. A new FB element (FB034) was obtained by multipartite assembly using *P. chrysogenum paf* promoter (FB029), the transcriptional unit for *sca* expression (FB032) and the *paf* terminator (FB030). FB034 was assembled with the *hph*-resistant cassette (FB003) to obtain the binary vector FB038. Both binary vectors for *sca* disruption and overexpression were transformed into *A. tumefaciens* AGL-1 by electroporation.

Fungal transformation of *P. digitatum* CECT 20796 with pGKO2_Δ*sca* and FB038 vectors was performed following the *A. tumefaciens*-mediated transformation (ATMT) protocol previously described [48,49]. Sca ectopic transformed strains were selected in 25 μg/mL hygromycin B (Invivogen, San Diego, CA, USA). On the other hand, homologous transformants were initially pre-screened in 25 μg/mL hygromycin B as a positive selection and subsequently in 25 μM 5-fluoro-2′-deoxyuridine (F2dU) (Merck, Darmstadt, Germany) as a negative selection. All transformant strains were confirmed by PCR using genomic DNA as described previously [41] (Supplementary Figures S3 and S4). The size and presence of DNA amplicons were determined by 1% agarose gel electrophoresis.

### 2.5. Protein Production and Purification

Sca was purified from the supernatant of *P. digitatum* Sca^op^ strains (10^6^ conidia/mL) grown in *P. digitatum* minimal medium (PdMM) [50] after 11 days of growth at 25 °C with strong aeration. Cell-free supernatant was collected by centrifugation and dialyzed (2K MWCO, Sigma-Aldrich, St. Louis, MO, USA) against 20 mM phosphate buffer at pH 6.8. Given the predicted chemical properties of Sca (pI = 4.54), the dialyzed solution was applied to an AKTA Purifier system equipped with a 6 mL RESOURCE Q column (GE Healthcare, Chicago, IL, USA) equilibrated in the phosphate buffer. Elution was set with a linear NaCl gradient from 0 to 1 M in the same buffer. Surprisingly, Sca protein was not adsorbed in the resin and was present (as the major protein) in the flow-through after chromatography (Supplementary Figure S5). Thus, the Sca-containing flow-through was collected, dialyzed against Milli-Q water and lyophilized. Protein concentration was determined spectrophotometrically (A_280_) considering the Sca molar extinction coefficient (Ɛ_280_ = 2.43). The purity was monitored by SDS-PAGE using SDS-16% polyacrylamide gels calibrated with pre-stained protein size-standard SeeBlue^®^ and Coomassie blue staining. AfpB protein production, purification, and quantification were achieved as previously described [16].

### 2.6. Antimicrobial Activity Assays

The antifungal activity of Sca was evaluated with two different growth inhibition assays. A final concentration of 10^5^ conidia/mL from *P. digitatum* CECT 20796 was inoculated in 100 mL flasks containing 25 mL 1/4 diluted PDB supplemented with different concentrations of Sca (6 µg/mL) or AfpB (0.032 and 0.065 µg/mL) proteins. To evaluate potential synergism between Sca and AfpB, combinations of both proteins were added simultaneously (6 and 0.032 µg/mL or 6 and 0.065 µg/mL of Sca and AfpB, respectively). Cultures were grown under strong aeration at 25 °C for 48 h. After this, mycelia were recovered, filtered, washed and paper-dried, and (wet weight) biomass was measured.

Additional growth inhibition assays with Sca, AfpB and PeAfpA against the chosen fungi (Supplementary Table S3) were performed in 96-well flat bottom microtiter plates (Nunc, Roskilde, Denmark) in a total volume of 100 µL as described previously [29]. Synergy assays in 96-well microtiter plates were conducted as follows: 25 µL of two different 4× concentrated proteins were mixed in the same well with 50 µL of *P. digitatum* conidia (5 × 10^4^ conidia/mL) in 1/10 diluted PDB containing 0.02% (*w/v*) chloramphenicol to avoid bacterial contamination. Both protein concentrations in these experiments were: 2 and 32 µg/mL for Sca; from 0.03 to 16 µg/mL for AfpB. In all cases, plates were statically incubated for 4 days at 25 °C, and growth was determined daily by measuring the optical density at 600 nm (OD_600_) using a FLUOstar Omega plate spectrophotometer (BMG labtech, Orlenberg, Germany). Data are expressed as OD_600_ means ± standard deviation (SD) of three replicates and dose–response curves were generated from measurements after 72 h.

Antibacterial assays were carried out as described previously [51]. Different experiments were repeated at least twice. The minimum inhibitory concentration (MIC) is defined as the peptide concentration that completely inhibited growth in all experiments.

### 2.7. Fruit Infection Assays

In vivo analyses were performed by infecting non-treated, mature, freshly-harvested orange fruits (*Citrus sinensis* L. Osbeck cv Navel and Lanelate) with different *P. digitatum* strains following previous protocols [43]. Three replicates of five fruits were inoculated at four wounds around the equator with 5 µL of conidial suspensions (10^4^ conidia/mL). At different days post-inoculation (dpi), each inoculated wound was scored for specific green mold infection symptoms. Data were calculated as the means and SD of the percentage of infected wounds. Moreover, tissue samples (discs of 5 mm in diameter around the inoculation site) at 1, 2, 3, 4 or 7 dpi for quantitative RT-PCR were collected, crushed and frozen at −80 °C to be used for RNA extraction.

### 2.8. Statistical Analysis

Differences in protein activities were determined using the one-way analysis of variance (ANOVA) and Tukey’s honestly significant difference (HSD) test. Statistical significance was referred for *p* value < 0.05. Analyses were done using STATGRAPHICS Centurion XVI Version 16.1.17 and Microsoft Excel 2016 software (Real Statistics Resource Pack, http://www.real-statistics.com/).

## 3. Results

### 3.1. A Novel Small Cysteine-Rich and Anionic (Sca) Protein Is Abundantly Produced and Secreted by P. digitatum

**Figure 1:**
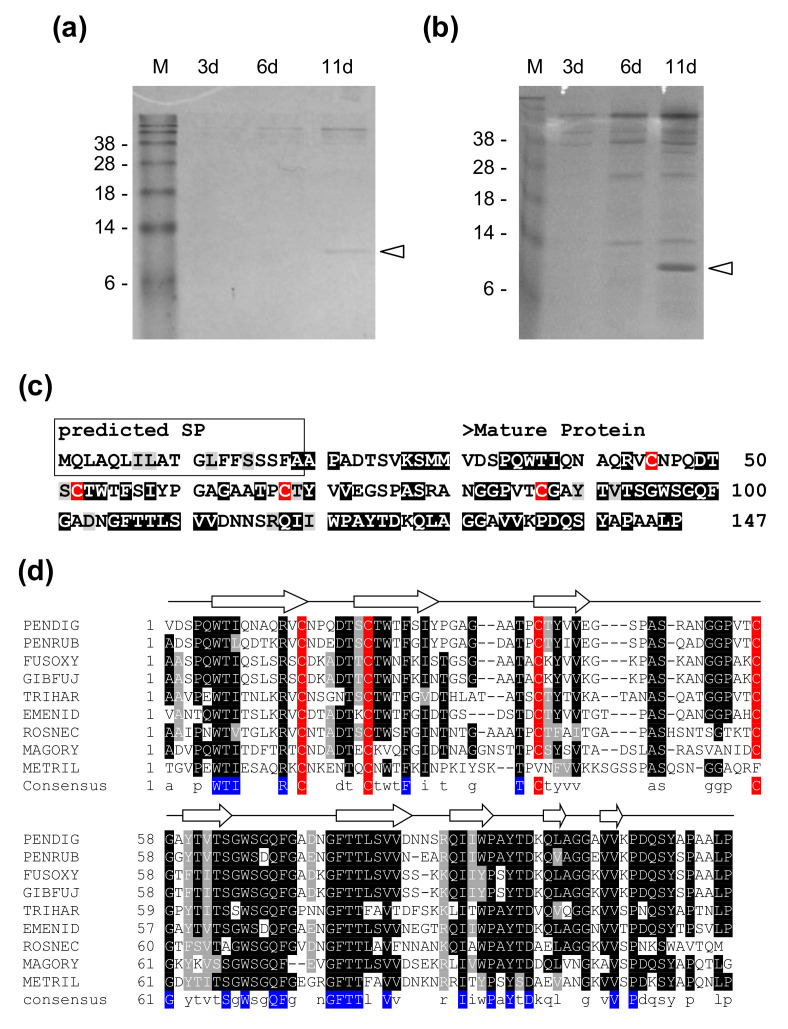
Production and identification of the Secreted Cysteine-rich Anionic (Sca) protein from *P. digitatum* CECT 20796 strain. (**a**) SDS-PAGE of non-concentrated supernatants of *P. digitatum* grown in potato dextrose broth (PDB) for 3, 6 and 11 days. Sca is marked by an arrow. (**b**) SDS-PAGE of the same supernatants 10× concentrated. M: SeeBlue ^®^ pre-stained protein standard. (**c**) Amino acid sequence of the Sca protein. Predicted signal peptide (SP, framed) and small pro-sequence are absent in the mature protein. Highly conserved and conserved amino acids are shadowed in black and in gray, respectively, and are concluded from the alignment in Supplementary Figure S2. (**d**) Amino acid sequence alignment of chosen Sca homologs from different filamentous fungi. Arrows represent predicted secondary structural elements of Sca from *P. digitatum*. Conserved cysteine residues are shadowed in red. Extremely conserved amino acids are shadowed in blue in the consensus sequence. Highly conserved amino acids are shadowed in black and conserved amino acids are shadowed in gray. Abbreviations: PENDIG, *Penicillium digitatum*; PENRUB: *Penicillium rubens*; FUSOXY, *Fusarium oxysporum*; GIBFUJ, *Gibberella fujikuroi*; TRIHAR, *Trichoderma harzianum*; EMENID, *Emericella nidulans*; ROSNEC, *Rosellinia necatrix*; MAGORY, *Magnaporthe oryzae*; METRIL, *Metarhizium rileyi*. An extended version of this alignment can be seen in Supplementary Figure S2.

In an attempt to detect the antifungal protein AfpB in the culture supernatant of *P. digitatum* CECT 20796, a faint band of a protein of about 12 kDa was detected by SDS-PAGE after 11 days of growth in PDB (Figure 1a), which was more evident after tenfold concentration of the supernatant samples (Figure 1b). In order to identify this protein, a MALDI-TOF/TOF analysis was performed and the results revealed that this protein was encoded by the PDIG_23520 gene. We named it Sca (Secreted Cysteine-rich and Anionic), an anionic protein (pI = 4.54) with a molecular mass (MM) of 12,205.4, 117 amino acids and 4 cysteines (Supplementary Figure S1). A SP of 19 residues was identified at the N-terminus, indicating its processing and secretion, followed by a pro-sequence of 11 amino acids which is missing in the mature protein (Figure 1c and Supplementary Figure S1). BLASTP analyses revealed the presence of Sca homologs in a wide range of filamentous ascomycetes. Based on protein sequence alignments, two clear domains can be distinguished in the mature Sca protein: (i) a more variable N-terminal domain that contains four conserved cysteines and two likely disulphide bridges, and (ii) a much more conserved C-terminal domain, which is rich in aromatic residues (Figure 1d, Supplementary Figures S1 and S2). In the N-terminal domain, four cysteines were highly conserved in all protein sequences except for the ones from *Metarhizium rileyi* and *Pochonia chlamydosporia* (Figure 1d and Supplementary Figure S2). Secondary structure prediction based on the full sequence alignment showed that Sca is a beta-stranded protein with eight β-sheet structural motifs (Figure 1d). Finally, Pfam domain searches did not reveal any known functional motif for Sca. Our results revealed the identification of a novel and highly-conserved protein not described to date and with an unknown function.

### 3.2. Gene Expression of Sca (PDIG_23520) and Its Comparison with afpB (PDIG_68840)

**Figure 2:**
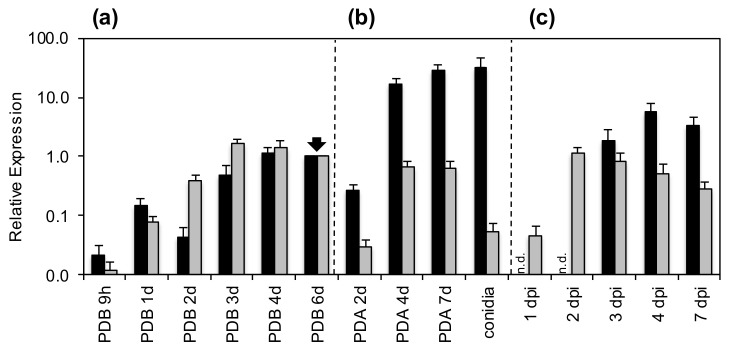
Relative expression of *afpB* and *sca* genes. Relative expression of *P. digitatum afpB* (black bars) and *sca* genes (gray bars) over different times of growth in: (**a**) liquid medium (PDB), (**b**) solid medium (potato dextrose agar (PDA)) and conidia and (**c**) during infection of citrus fruits. Gene expression for each condition was normalized independently to the expression at day 6 in PDB, marked by a black arrow. n.d, expression of *afpB* not detected in these infection samples. dpi, days post-inoculation. Bars show the means ± standard error (SE) of three technical replicates. Note the logarithmic scale for Y axis.

In order to further characterize this novel protein, the relative expression pattern of its encoding gene PDIG_23520 was determined during fungal axenic growth and infection, and compared to that of PDIG_68840, which encodes the previously reported AfpB (Figure 2). The expression pattern of both genes was similar during submerged growth in PDB, reaching the highest value of induction after 3-4 days of growth (Figure 2a). In contrast, *afpB* is much more induced than *sca* during aerial growth on PDA plates, concomitant with conidiogenesis, and the highest amount of *afpB* mRNA is found in quiescent conidia with levels more than 10 times above the reference condition (Figure 2b), as previously described [22]. In contrast, the amount of *sca* mRNA in conidia is more than 10 times below the reference condition. Remarkably, upon infection (Figure 2c), the *sca* expression pattern indicates a very early induction (from the first dpi) and a later decline, which qualitatively differs from the gene expression pattern of *afpB* which was not detected until 3 dpi, and from this point onwards, the relative level of mRNA remained approximately constant. It must be noted that maceration symptoms appeared from 3 dpi. These results would suggest a relevant role of Sca during fruit infection in its initial stages.

### 3.3. Null Mutants and Overproducers Do Not Show Phenotypic Differences with Parental Strain during Axenic Growth

**Figure 3:**
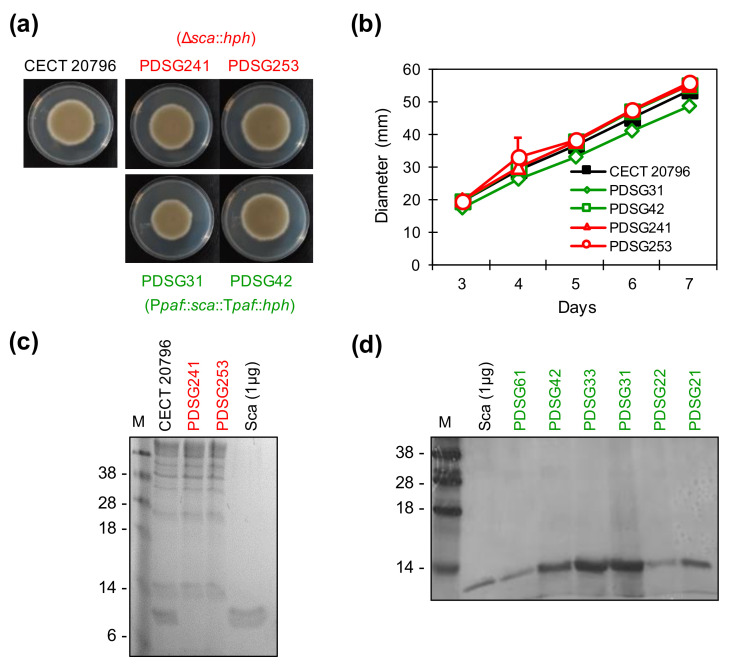
Characterization of different *Penicillium digitatum sca* null and overproducer strains. (**a**) Images of PDA plates after 5 days of growth of *P. digitatum* CECT 20796, null Δ*sca* strains (PDSG241 and PDSG253, in red) and Sca^op^ strains (PDSG31 and PDSG42, in green). (**b**) Colony diameter on PDA plates from 3 to 7 days of the same strains represented as the mean ± SD of three replicates. (**c**) SDS-PAGE of 10× supernatants of *P. digitatum* CECT 20796 and null Δ*sca* strains (PDSG241 and PDSG253) grown in PDB for 11 days. Pure Sca protein (1 µg) was used as control. Note that the band corresponding to the Sca protein disappeared in these mutants. (**d**) SDS-PAGE of 8-day non-concentrated *P. digitatum* minimal medium (PdMM) supernatants of different *P. digitatum* Sca^op^ strains compared with 1 µg of pure Sca protein. M: SeeBlue ^®^ pre-stained protein standard.

Null (Δ*sca*) and overproducer (Sca^op^) mutants were generated in order to study the biological role(s) of *sca* gene in *P. digitatum*. To obtain the null mutants, *sca* was replaced with the *hph* cassette as a positive selection marker for hygromycin resistance by homologous recombination (Supplementary Figure S3). The binary vector pGKO2_Δ*sca* obtained to delete the *sca* gene also contains the *HSVtk* gene used as a negative selection marker to discard ectopic insertions, as previously described [22,41] (Supplementary Figure S3a). Six independent Δ*sca* transformants were obtained and confirmed by PCR using a set of distinct primer combinations located inside and outside the constructs (Supplementary Figure S3b and Supplementary Table S2). The FB modular cloning technology recently described [47,48] was applied to generate Sca^op^ strains aimed at overproducing the Sca protein, as described in Materials and Methods and Supplementary Figure S4. We aimed to generate Sca^op^ strains by using the *paf*-based expression system [50], which contains the *paf* promoter and terminator sequences from *P. chrysogenum*, and has been demonstrated to work efficiently for the biotechnological production of small CRPs with antifungal activity, including AfpB [16,29,50]. Molecular characterization of six independent overproducers was confirmed by PCR using different primer sets (Supplementary Figure S4b,c and Supplementary Table S2). Two independent strains of Δ*sca* and Sca^op^ transformants were selected to characterize their phenotypic behavior, and results showed no major phenotypical differences under axenic culture on PDA plates (Figure 3a,b), indicating that gene deletion or protein overproduction has no influence on *P. digitatum* growth ability. SDS-PAGE analysis of tenfold concentrated supernatants obtained from CECT 20796 and the two Δ*sca* strains phenotypically characterized (PDSG241 and PDSG253) confirmed the lack of Sca protein band in the mutants grown on PDB (Figure 3c). In contrast, Sca^op^ transformants produced large amounts of Sca after 8–10 days of growth in PdMM [50], which were clearly visible and highly abundant even in the non-concentrated culture supernatants (Figure 3d).

### 3.4. The Purified Sca Does Not Have Antimicrobial Activity In Vitro but Enhances the Antifungal Activity of AfpB

Protein Sca was purified from culture supernatant of Sca^op^ PDSG31 grown for 11 days in PdMM. Due to predicted chemical properties (pI = 4.54), anionic exchange chromatography procedures were applied to previously dialyzed Sca-rich supernatant. However, the protein was not adsorbed in the resin and was eluted in the flow-through with high purity, free of most of the high molecular weight contaminants (Supplementary Figure S5).

**Figure 4:**
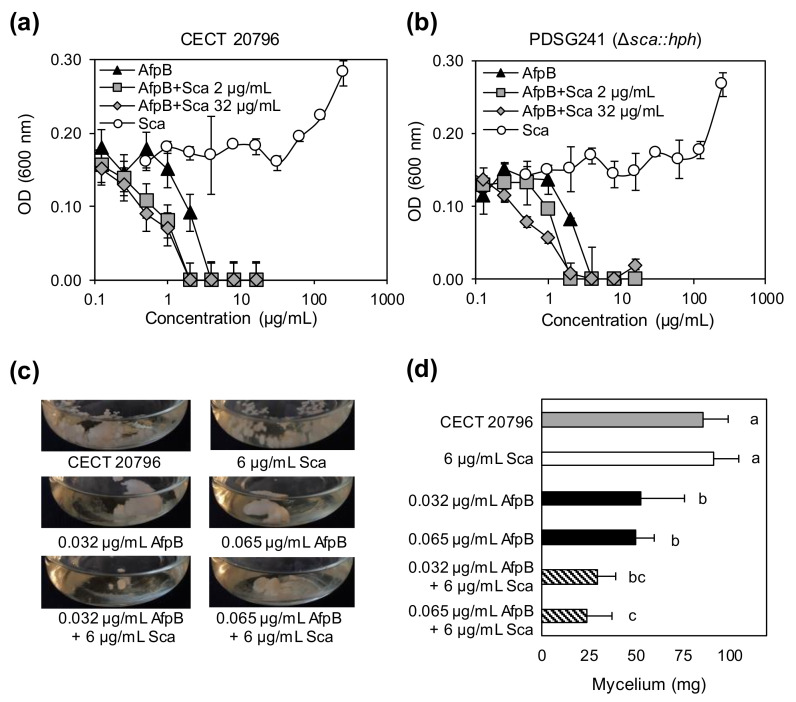
In vitro antifungal assays of the Sca/AfpB proteins against *P. digitatum*. (**a**) Dose–response curves showing in vitro antifungal activity of AfpB and Sca proteins against *P. digitatum* CECT 20796. Note the absence of antifungal activity in Sca and improvement of antifungal activity of AfpB due to addition of the Sca protein at different concentrations. (**b**) Dose–response curves showing in vitro antifungal activity of AfpB and Sca proteins against *P. digitatum* PDSG241 Δ*sca* strain. Similar details as in (**a**). (**c**) Representative images of growth inhibition assays in flasks containing different amounts of proteins and protein combinations as indicated. (**d**) Graph representing the weight of collected mycelia from (**c**). Bars show means ± standard deviation (SD) of biological triplicates. Letters show significant differences among the treatments (ANOVA and Tukey’s honestly significant difference (HSD) test, *p* < 0.05).

The Sca protein is a relatively small (12 kDa), anionic, secreted CRP with a β-strand-rich predicted secondary structure without a known function. Given all these features, we first aimed to test a possible role of Sca as a novel anionic AMP. For this, in vitro inhibitory assays were performed against several filamentous fungi, including its parental fungus, and the model yeast *S. cerevisiae*, using the antifungal proteins AfpB and PeAfpA as controls (Figure 4 and Supplementary Table S3). Antibacterial activity was assayed against the Gram-negative *E. coli* and the Gram-positive *B. subtilis*. Experiments performed in 96-well plates, with a Sca concentration ranging 0.5-256 µg/mL, revealed that Sca did not show any in vitro inhibitory effect against any of the microorganisms tested, including the parental (Figure 4a) or null Δ*sca* (Figure 4b) strains under conditions in which AfpB and PeAfpA were active (Supplementary Table S3). Remarkably, high concentrations of Sca (over 128 µg/mL) enhanced the biomass of both *P. digitatum* strains. Unexpectedly, the addition of different amounts of Sca improved the antifungal activity of AfpB (Figure 4a,b), lowering twofold its MIC value of 4 µg/mL, and this effect was also similarly observed in both strains.

In parallel, other experiments were conducted in which the fungus was grown in large culture volumes undergoing strong aeration using Erlenmeyer flasks (Figure 4c). Results revealed that addition of AfpB at very low sub-inhibitory concentrations (0.032 and 0.065 µg/mL) significantly reduced *P. digitatum* biomass production. Under these conditions, AfpB shows a higher apparent antifungal activity than in 96-well plates, in which the MIC value of 4 µg/mL was determined [16]. Next, we tested the combination of Sca and AfpB to show that Sca at 6 µg/mL also enhanced the activity of AfpB at 0.065 µg/mL when added simultaneously, resulting in a significant decrease in fungal biomass (Figure 4d), and confirming the results obtained in 96-well plates (Figure 4a).

### 3.5. Sca Enhances the Virulence of P. digitatum to Citrus Fruits and Compensates the Antifungal Effect of AfpB Upon Infection

**Figure 5:**
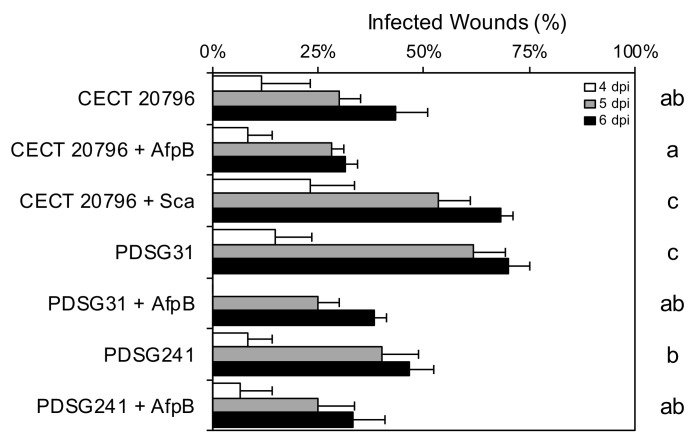
Effect of *P. digitatum* Sca and AfpB proteins on infection assays caused by different *P. digitatum* strains. Incidence of infection of wounds inoculated with 10^4^ conidia/mL of different *P. digitatum* strains (parental CECT 20796, Sca^op^ PDSG31 and Δ*sca* PDSG241) alone or in the presence of 100 µg/mL of AfpB or 180 µg/mL of Sca. Data indicate the percentage of infected wounds (mean values ± SD of three replicates of five oranges) at each day post-inoculation (dpi). Different letters show statistical significance of the infection incidence compared to the control sample at 6 dpi (ANOVA and Tukey’s HSD, *p* < 0.05).

**Figure 6:**
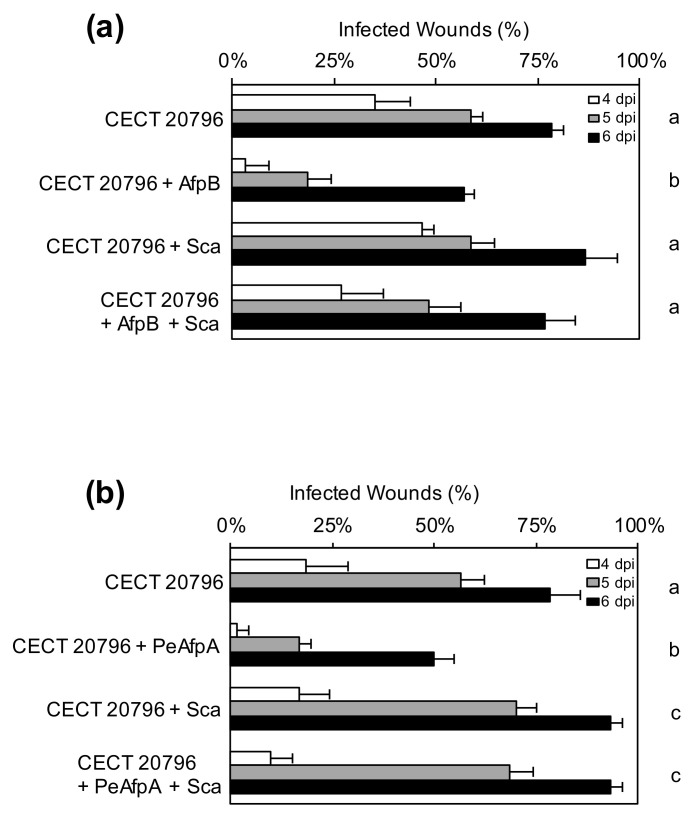
Effect of different proteins and protein combinations on the infection caused by *P. digitatum* CECT 20796 strain. (**a**) Incidence of infection of wounds inoculated with 10^4^ conidia/mL of parental CECT 20796 strain alone or in the presence of 100 µg/mL of AfpB, 180 µg/mL of Sca or combinations of both proteins. Data indicate the percentage of infected wounds (mean values ± SD of three replicates of five oranges) at each dpi. Different letters show statistical significance of the infection incidence compared to the control sample at 6 days (ANOVA and Tukey’s HSD, *p* < 0.05). (**b**) Incidence of infection of wounds inoculated with 10^4^ conidia/mL of parental CECT 20796 strain alone or in the presence of 100 µg/mL of PeAfpA, 180 µg/mL of Sca or combinations of both proteins. Other details as in (**a**).

The early expression pattern of the Sca-encoding gene at the onset of fruit infection suggested a role of this protein during infection caused by *P. digitatum* (Figure 2c). Therefore, the possible role of Sca during infection of citrus fruit was evaluated. Addition of purified Sca to the *P. digitatum* inoculum always enhanced fungal virulence, although this increase was not statistically significant in all assays (Figure 5 and Figure 6). In accordance with the enhanced virulence with purified protein, Sca^op^ PDSG31 was also more virulent than the parental strain. Taken together, both results indicate that Sca improves *P. digitatum* virulence in vivo. However, virulence of the Δ*sca* mutant (PDSG241) did not significantly differ from that of the parental strain and, therefore, the canonical definition of a virulence gene or an effector was not fulfilled.

AfpB was previously reported to show poor or no protective effect against *P. digitatum* on oranges from the Navelina variety [29]. In the current study, AfpB showed a slightly protective effect against infection when using Navel or Lanelate varieties (Figure 5 and Figure 6a). Addition of AfpB to the Sca^op^ PDSG31 or the Δ*sca* PDSG241 inocula also resulted in a reduction in the incidence of infection to levels similar to those of the parental CECT 20796.

A modulating effect of Sca over AfpB was shown when both proteins were added simultaneously to *P. digitatum* inoculum, since the presence of Sca counteracted the protective effect of AfpB against infection (Figure 6a). A similar modulating effect was shown with PeAfpA from *P. expansum*, another AFP previously reported to efficiently control *P. digitatum* infection in vivo [29]. Addition of PeAfpA to *P. digitatum* inoculum had a protective effect that was compensated by the addition of Sca (Figure 6b). These experiments also confirmed that pure Sca improved incidence of infection, supporting results obtained in the previous experiments (Figure 5). Therefore, Sca counteracted the protective effect exerted by the homologous AfpB (Figure 6a) or the heterologous PeAfpA (Figure 6b) on fungal infections.

## 4. Discussion

In this study, we described the identification and production and initiated the characterization of a novel and highly conserved protein within a wide range of ascomycete fungi with an unknown function. The Sca protein is a small, stable, CRP with a β-strand-rich predicted secondary structure and anionic nature. The presence of SP and pro-sequence is indicative of post-translational processing and confirmed its character as a secretory protein. All these features are compatible with antimicrobial peptides and proteins. The production of anionic antimicrobial peptides and proteins has been previously described in many organisms [52]. Examples of these are maximin H5 from amphibian skin [53]; iturins from *Bacillus* genus [54], or AfusinC, an anionic fungal defensin from *Aspergillus fumigatus* with bactericidal effects [55]. In an attempt to find the functional role of Sca, putative antimicrobial activity of this protein was evaluated. However, antimicrobial assays against different fungi, yeast and bacteria (Supplementary Table S3) suggest that Sca does not appear to be an antimicrobial agent, since neither antifungal nor antibacterial activity was detected under conditions at which other AFPs (AfpB or PeAfpA) were inhibitory [16,29].

Null and overexpression *sca* mutants show that this gene is dispensable for fungal vegetative growth and fruit infection, and that protein overproduction does not negatively affect the fitness of the producer strains, including pathogenicity or virulence. Sca overproduction was achieved by using a *paf* promoter-driven expression cassette from *P. chrysogenum*, which was previously used for the successful production of some AFPs. For instance, this cassette was applied to produce modified versions of the PAF protein from *P. chrysogenum* [50], NFAP and NFAP2 from *Neosartorya fischeri* [50,56], AfpB from *P. digitatum* [16], PAFB from *P. chrysogenum* [19] or PeAfpA, PeAfpB and PeAfpC from *P. expansum* [29]. However, in this study, we report, for the first time, the successful use of this *paf*-based expression system for the high-yield production of a non-AFP protein in a filamentous fungus. Additionally, Sca has also been efficiently produced in *N. benthamiana* using a virus-based expression system [28], which indicates that Sca is a stable protein that can be easily produced in different systems and accumulates in large amounts in different organisms. As such, the possibility of using Sca as a fusion carrier for the high-yield production of heterologous proteins will be explored in the near future.

Beyond their antifungal activity, some AFPs have been described to play different biological roles or have alternative functions [57,58,59,60,61]. However, the biological role of *afp* genes in filamentous fungi is not well understood, and little is known about functional interactions with other biomolecules in the producer fungi. In this study, we report, for the first time, that AfpB shows stronger antifungal activity when tested in large volume cultures undergoing strong aeration compared to small-scale experiments performed statically in 96-well plates. This unexpected observation is, at present, under investigation in our laboratory to determine the causes of this previously unknown behavior, which could be related to the biological role(s) and/or mode of action of this AFP in the parental fungus. Additionally, we show that Sca improves AfpB inhibitory activity in vitro, whereas it compensates the antifungal effect of AfpB in vivo, indicating a dual effect that would suggest a sort of functional interaction between these two proteins. Immunoprecipitation experiments were carried out in order to analyze possible Sca interaction with AFPs, which could be due to the presence of opposite net charges, but no co-immunoprecipitation was observed after testing several experimental conditions (data not shown). It could be possible that the functional interaction between Sca and AfpB/PeAfpA does not require any physical interaction. Further research will be done in order to elucidate the putative mechanisms by which Sca and AfpB would functionally interact in their producer fungus.

Based on gene expression, CRP nature, secretion and protein size, it could be hypothesized that Sca might be either (i) an effector of virulence, or (ii) a sort of “immune” mechanism to protect *P. digitatum* from the self-inhibitory activity of AfpB. Protein fungal effectors in plant pathogens modulate the interaction between the fungus and its plant host by either killing the host cell or protecting the fungus from the plant defense system, thus enhancing its virulence [62]. Two independent approaches (the addition of pure protein to the fungal inoculum and the increased virulence of Sca^op^ strains) indicate that Sca enhances *P. digitatum* virulence. Effectors are usually small secreted proteins (<300 amino acids), very rich in cysteine residues involved in disulphide bridges and are induced at early stages of infection, features that are also compatible with Sca. These proteins also contain specific Pfam domains as Lysin motifs (LysM), carbohydrate-binding protein modules, which play an important role in the virulence of several plant pathogens [63,64,65]. No Pfam domains or presence of LysM or chitin-binding domains indicative of effector function were found in Sca. Another characteristic common to many fungal effectors is the reduction in or lack of virulence of the corresponding null mutants [66,67]. However, our null Δ*sca* strains did not show negative effects on virulence, similar to some deletion LysM mutants for which no significant changes on the incidence of infection were observed [68]. Presence of Sca homologs in non-phytopathogenic fungi (Figure S2) and absence in other phytopathogens (such as *Penicillium italicum* or *P. expansum*) could also indicate that Sca does not have a role as a fungal effector. Therefore, this protein does not fulfill some of the “canonical” properties of an effector, although it enhances fungal virulence. Therefore, our discovery of Sca supports either a redefinition of the currently accepted concept of effector protein or the possibility that some effectors might be redundant and not detectable by single gene deletions.

Our first working hypothesis was that Sca might be an immunity factor to protect *P. digitatum* against the self-inhibition of AfpB, based on the strong opposite net charges of both proteins that could block the cationic character of AfpB. However, our efforts to demonstrate a physical interaction between Sca and AFPs through immunoprecipitation were not successful. In this study, it can be observed that Sca increases *P. digitatum* growth in vitro and virulence in vivo and compensates and overcomes the antifungal effect of both AfpB and PeAfpA during citrus fruit infection, which is the natural niche of this fungus. This fact may be explained as a consequence of the increased virulence driven by Sca, contrary to the results observed in in vitro assays where AfpB activity was improved by addition of pure Sca. These observations confirm that results observed in in vitro assays do not always correlate with the in vivo effects, emphasizing the need for in vivo assays to better characterize protein activities and putative interactions.

Even though our experimental data do not fully support the proposed alternatives on the putative roles of Sca as an effector of virulence or an immunity factor, both alternatives remain open. Future research will focus on carrying out different experimental setups to uncover the functional role of Sca in its producer fungus.

## 5. Conclusions

In summary, our work presents a novel protein that enhances fungal virulence in vivo and fungal growth in vitro, and is able to modulate the inhibitory activity of AFPs. Different assays were accomplished to reveal the functions of this protein. However, our results excluded the role of Sca as an antimicrobial agent or as a canonical effector. Further studies will be conducted to unravel the biological and functional role(s) and the relevance of this highly conserved protein in filamentous ascomycete fungi.

## Supplementary Materials

The following are available online at https://www.mdpi.com/2309-608X/6/4/203/s1, Figure S1: Identification of Sca protein in *P. digitatum* CECT 20796 strain; Figure S2: Amino Acid sequence alignment of *P. digitatum* Sca and putative homologous from different filamentous fungi; Figure S3: Generation of null Δ*sca* strains; Figure S4: Generation of overproducer strains (Sca^op^) using the FungalBraid approach; Figure S5: SDS-PAGE analysis of fractions obtained after anion exchange chromatography. Table S1: qRT-PCR primers used in this work; Table S2: PCR primers used to generate different constructs and to verify the transformants of *sca* gene; Table S3: Minimum inhibitory concentration (MIC) values (µg/mL) of Sca, AfpB and PeAfpA against the microorganisms tested.
