# Supplementary material for: A Novel Secreted Cysteine-Rich Anionic (Sca) Protein from the Citrus Postharvest Pathogen Penicillium digitatum Enhances Virulence and Modulates the Activity of the Antifungal Protein B (AfpB)
Source: J Fungi (Basel). 2020 Oct 2;6(4):203. doi: 10.3390/jof6040203 (PMC7711571; doi:10.3390/jof6040203)
Supplement: Supplementary file 1 [file jof-06-00203-s001.pdf]

Supplementary Figures

Journal of Fungi

A novel Secreted Cysteine-rich Anionic (Sca) protein from the citrus postharvest pathogen Penicillium digitatum enhances virulence and modulates the activity of the Antifungal Protein B (AfpB)

Sandra Garrigues †, Jose F. Marcos, Paloma Manzanares, and Mónica Gandía *

Figure S1

>tr|K9G4Z7|K9G4Z7_PEND2 Uncharacterized protein OS=*Penicillium digitatum* (strain PHI26 / CECT 20796) GN=PDIG_23520 PE=4 SV=1

Mature Protein Parameters pI 4.54; MW 12205.4; Number of amino acids 117

Signal Peptide | Mature protein: Cysteine domain | Conserved domain

```
                              -  @      +     -   @ @             @  -     +      @     @   @  -  @       -   +   @  @ -+        + -  @
MQLAQLILATGLFFSSSFAAPADTSVKSMMVDSPQWTIQNAQRVCNPQDTSCTWTFSIYPGAGAATPCTYVVEGSPASRANGGPVTCGAYTVTSGWSGQFGADNGFTTLSVVDNNSRQIIWPAYTDKQLAGGAVVKPDQSYAPAALP
--HHHHHHHHHHHH-------------------EEEEEEEEEE-----EEEEEEEEE--------EEEEEE----------------EEEEE-----------EEEEEEEE----EEEEE-----EE----EE------------
```

N-terminal identification:

VDSP
SPQ

Mass Spectrometry Peptides:

MVDSPQWTIQ
VDSPQWTIQNAQR
DSPQWTIQNAQR
SPQWTIQNAQR
VCNPQDTSCTWTF
SIYPGAGAATPCTYVVEGSPASR
YVVEGSPASR
QIIWPAYTDK
QLAGGAVVKPDQSYAPAALP
QLAGGAVVKPDQSYAPA
QLAGGAVVKPDQSY

Extremely conserved (>93%), highly conserved or conserved amino acids in homologous proteins of filamentous fungi; conserved cysteines

Notes:
MS data: in blue, the ten most abundant peptides; in red, a minor peptide (0.1% of total signal 8385 / 5681121) that indicates another alternative processing.

Figure S1. Identification of Sca protein in *P. digitatum* CECT 20796 strain. Peptide mass fingerprinting (PMF) and N-terminal sequencing of the Sca protein from *P. digitatum* corresponding to the predominant 12 kDa band found in PDB supernatant. Peptides obtained by PMF covered 78 % of Sca primary sequence. Predicted signal peptide and small pro-sequence are absent in mature protein. Conserved cysteines are shadowed in red. Extremely conserved amino acids are shadowed in blue. Highly conserved amino acids are shadowed in black and conserved amino acids are shadowed in gray.

```
PENDIG        1 --MQLAQLILATGLFFSSSFAAP--ADT-SVKSMMVD-SPQWTIQNAQRVCNPQDTSCTWTFSIYP-GAG
PENRUB        1 --MQLTQLLLATGLLASSAFAAP--ADT-SAKSMMAD-SPQWTLQDTKRVCNDEDTSCTWTFGIYP-GAG
FUSOXY        1 --MKFTAALIA-LVSATGAVAAP--APA-DSVSMMAA-SPQWTIQSLSRSCDKADTTCTWNFKIST-GSG
GIBFUJ        1 --MKFTAALIA-LVSATGAVAAP--APA-DSVSMMAA-SPQWTIQSLSRSCDKADTTCTWNFKINT-GSG
FUSKUR        1 --MKFSLAFVAALLSATNVAAAPS-ADP-NAVSMMAA-TPQWTIQNMQRTCDKGDTTCTWNFKINT-GKG
TRIHAR        1 --MQITKSLIATLFAASTAFAAPTPAD----KSMMAA-VPEWTITNLKRVCNSGNTSCTWTFGVDTHLAT
ASPCAL        1 MKLSLSTLLVTALSAG-SALAAPAPAES---KSMMVA-NTQWTITNMKRVCNTADTKCTWTFGINN--GA
EMENID        1 MKLSLNLLLATLSLSS-TAFAAPSSA-----KSMMVA-NTQWTITSLKRVCDTADTKCTWTFGIDT--GS
TALISL        1 --MQLTQILVLLA-ASSTTLAAP--IG--NTKSMMAA-SPEWTIKSLKRVCTADNSSCTWTFGIYD-GAD
ASPVER        1 --MKFLTQAILAALACTPALAAP--A---KTKSMMIK-ESEWTIESLKRECNDDDSKCTWTFGINP-GSG
VALMAL        1 --MQFSTLLVSALLSA-TGMAAPSQPRA-GAVSMMAD-ASTWTITGLSRVCNAADTSCTWSFGINN--GT
GIBMON        1 --MQFSLACLA-AILATSVSAAP--AP---AVNMMAA-SPQWTIENMQRSCAKDDSSCTWNFKIDT-HKG
TRILON        1 --MQLTKTLFATLLAASTAFAAPTPAD----KSMMAA-VPEWTITDMKRVCNKANTECTWTFGVDTHLAA
HYPJEC        1 ------------LLAASTAFAAPTPAD----KSMMAA-VPQWTITNMKRVCNKANTECTWTFGVDTHLAA
GIBZEA        1 --MKFSAAVFT-FIAAAGVSAAP--AEE-KAVNMMAA-APQWTIRDAKRYCRSDDSICNWKFGIDT-GNG
POCCHL        1 --MQLTQTIVAALFAASSVVAAPAPSE----KSMMTTGVPQWTIEGVKRVCDGADNQCNWTFKINPKIYS
PENOXA        1 --MQFTQVLLATLLAVPAALAAP--A---SGKSMMAQ-SPQWTIQNMKRVCAANDSSCTWTFGIYP-GSG
FUSPOA        1 --MKFTTSLFA-LAAATGAVAAPS-SPL-DAVSMMAA-TPMWTIESMQRTCDKPDTTCTWNFKINT-GSG
HYPVIR        1 --MQITKTLVATLFAASTAFAAPTPAD----KSMMAA-VPEWTITNLKRVCNAGNTSCTWTFGVDTHLAT
TOLCAP        1 --MQLTKTLVAAIVAASAATAAPTADK----KSMMAA-VPQWTIVNMTRICAKDDSDCIWSFGINTHLAD
METRIL        1 --MQLTQTIVAALFAASSVVAAPAQPE----KSMMTTGVPEWTIESAQRKCNKENTQCNWTFKINPKIYS
ROSNEC        1 --MQFTTAAIAAILSASSVFAAP------------AA-IPNWTVTGLKRVCNTADTSCTWSFGINTNTGA
VERLON        1 --MHFPTVAVFAALLSGSALAAPAAN----VKSMAVA-STTWTIENTRRACKAND--CTWTFSVNTGSSN
VERNON        1 --MHFPTVAVFAALLSGSALAAPAAN----VKSMAVA-STTWTIENTRRACKAND--CTWTFSVNTGSSN
COLORB        1 --MQITNIVLSALATAAAVSASPVAKR--ETKSMMAA-APEWTIEGFTRTCNAQDTSCDYSFSINTHTAA
ACRCHR        1 --MKFSVTALLTTLLATSALAAPAPEPATGVKSMATA-AT-WTIENMKRTCNKADTSCAWSFTINAGGAK
COLFRU        1 --MQITNIFLSALA-AAAISASPVEKR--DSKSMMAA-VPEWTIEKFTRSCNAEDTSCNYSFSINTHTAD
MAGORY        1 --MLFTTTILA--LATLTG-AVPTPAG--QAKSMVAD-VPQWTITDFTRTCNDADTECKVQFGIDTNAGG
MAGPOA        1 --MHFSKTALLSLLAAAVS-AAPVPE---EAQSMVAA-VPQWTITNFKRTCNAEDTSCTVEFGVDTHLA-
CERZEI        1 --MQFTTTALALLTAASASLASPVPDQ--AQNMMATS-TPNWTIEKFTRTCNTDDTLCNYTFGINLNNGQ
BEABAU        1 --MHFSKTLLTVAVAATATAAAP----------LEAR-DPDWIIQGLSRACDGQDSSCTWKFAINNQLTA

consensus     1   m  t  il  ll as   AaP        ksmmaa  p WTI  l R C   dt CtwtF i t

PENDIG       64 A--ATPCTYVVEG---SPASRAN-GGPVTCGAYTVTSGWSGQFGADNGFTTLSVVDNNSRQIIWPAYTDK
PENRUB       64 D--ATPCTYIVEG---SPASQAD-GGPVTCGGYTVTSGWSDQFGAENGFTTLSVVN-EARQIIWPAYTDK
FUSOXY       63 A--ATACKYVVKG---KPASKAN-GGPAKCGTFTITSGWSGQFGADKGFTTLSVVS-SKKQIIYPSYTDK
GIBFUJ       63 A--ATACKYVVKG---KPASKAN-GGPAKCGTFTITSGWSGQFGADKGFTTLSVVS-SKKQIIYPSYTDK
FUSKUR       65 T--PTPCKYVVKG---RPASQTN-GGPAKCGTFTITSGWSGQFGPGNGFTVLSVIS-SNKQIIWPGYTDK
TRIHAR       64 A---TSCTYTVK--ATANASQAT-GGPVTCGPYTITSSWSGQFGPNNGFTTFAVTDFSKKLITWPAYTDV
ASPCAL       64 A--NTPCTLVVNG---TPASQTN-GGPKTCGPYTVTSGWSGQFGPNNGFTTLAVVNQNTRQIVWPAYTDV
EMENID       62 D--STDCTYVVTG---TPASQAN-GGPAHCGAYTVTSGWSDQFGAENGFTTLSVVNEGTRQIIWPAYTDK
TALISL       62 A--STACTYVVTA---PNASEAN-GGPSDCGVYTVTSGWSGQFGPGNGFTTLSVVDNKKRQIIWPGYTDK
ASPVER       62 ---ATDCTYEVEG---SPASEAN-GGPVNCGPYTITSGWSDQFGPEEGFTTLSVVKEETREIVWPAYTDK
VALMAL       64 G--TTQCTEVVTG---SPASQTN-GGPATCGVYTVTSGWSGQFGAGNGFTTLAVVNYGAGLIVYPAYTDT
GIBMON       61 A--ATGCKYVVKG--SKASQ-RN-GGPVKCGDFTITSGWSGQFGPGNGFTTFSVVS-SKRQIVWPAYTDK
TRILON       64 A---TPCTYVVK--AAANASQAS-GGPVTCGPYTVTSSWSGQFGPNNGFTTFAVVDYAKKLITWPAYTDV
HYPJEC       54 A---TPCTYVVK--AAANASQAS-GGPVTCGPYTVTSSWSGQFGPGNGFTTFAVVDYAKKLITWPAYTDV
GIBZEA       63 K--PYECRHDVKG--PGASKKRS-AGPTTCGDFTVTSGWSDVFGADNGFTTLSVVSNSKRQIIWPAYTDK
POCCHL       65 K---TPVNFVTKRSGATPASQNN-GVAQNFGDYTITSGWSGQFGPGNGFTTLAVVDNKNRRITWPAYTDK
PENOXA       62 N--ATPCTLVVTG---QKASQSN-GGPVNCGDYTVTSGWSGQFGPGNGFTTLAVVNNKSRQIAYPAYTDK
FUSPOA       64 A--ATPCKYVVKA--SKNASQAN-GGPAKCGTFTITSGWSGQFGAGNGFTTLSVVS-SKKQIVYPSYTDK
HYPVIR       64 A---TSCTYVVK--ANANASQAS-GGPVTCGPYTITSSWSGQFGPNNGFTTFAVTDFSKKLIVWPAYTDV
TOLCAP       64 T---TDCKLDVA--GPG-APQAN-GGPSDCGNYTVTSGWSGQFGPGNGFTTLAVVDNKARLIAFPAYTDK
METRIL       65 K---TPVNFVVKKSGSSPASQSN-GGAQRFGDYTITSGWSGQFGEGRGFTTFAVVDNKNRRITYPSYSDA
ROSNEC       56 A--ATPCTFAITG---APASHSNTSGTKTCGTFSVTAGWSGQFGVDNGFTTLAVFNNANKQIAWPAYTDA
VERLON       62 ----TPCTFHTKSTG-SDLPSRANGEAQTCGPYTVTSSWSGQFGEGNGFTTLAVVNRSSKQIVWPAYTDK
VERNON       62 ----TPCTFHTKSTG-SALPSRANGEAQTCGPYTVTSSWSGQFGEGNGFTTLAVVNRSSKQIVWPAYTDK
COLORB       66 ---VTPCKFTTA--G-SP-ASRAAS-NAKCGAYTVTSGWSGQFGPDAGFTTFSVTA--QKLIVWPAYTDK
ACRCHR       67 ----TACSYNVKKSG-KTPASRSPNKGTDCGVYTVTSGWSGQFGEGNGFTTLSVVNNPKRLIAWPAYTDK
COLFRU       65 ---ATACAFTTT--G-SP-ASRAAS-NAKCGAYTVTSGWSGQFGPDAGFTTFSVTD--QKLIVWPAYTDK
MAGORY       63 NS-TTPCSYSVT--A-DSLASRASVANIDCGKYKVSSGWSGQF--EVGFTTLSVVDSEKRLIVWPAYTDD
MAGPOA       63 -A-ATPCSYVVN--A-ASRASRARVDDVTCGPYKVSSGWDGHWGDENGFTTWAVVDFAQRLIVWPAYTDK
CERZEI       66 --PATGCNYNAS--G-KP-ASRATYQNVQCGDFRIGSTWSGQFGEGEGFQTLSVVK--DRQIIYPAYTDK
BEABAU       58 P---TACTYVVKAAGGKPASRSK-GGPVQCGDFRVTSDWSGQF-VVGFTTFAVFDTKKNL-I YPAYNDD

consensus    71     T Ctyvv       as    ggp  CG ytvtSgWsgQFg  nGFTTl Vv    r IiwPaYtDk
```

```
PENDIG      128 QLAGGAVVKPDQSYAPAALP-
PENRUB      127 QVAGGEVVKPDQSYSPAALP-
FUSOXY      126 QLAGGKVVKPDQSYSPAALP-
GIBFUJ      126 QLAGGKVVKPDQSYSPAALP-
FUSKUR      128 QVASGKVVKPDQSYAPANLPQ
TRIHAR      128 QVQGGKVVSPNQSYAPTNLP-
ASPCAL      128 QLKGGKVVKPDQAYTPAALP-
EMENID      126 QLAGGNVVTPDQSYTPSVLP-
TALISL      126 QLEGGNVVTPDQSYAPTALP-
ASPVER      125 QVDGGEVVVPDQSYTPQILP-
VALMAL      128 QVAGGAVVTPDQSYTPQALP-
GIBMON      124 QVQSGKVVKPDQSYTPANLPN
TRILON      128 QVQDGKVVTPNQSYAPTTLP-
HYPJEC      118 QVQDGKVVTPNQSYAPTTL--
GIBZEA      128 QLAGAKIVKPDQSYAPASLPK
POCCHL      131 QVAGGKVVSPDQSYAPQNLP-
PENOXA      126 QVEGGNVVKPDQSYPPYALP-
FUSPOA      128 QLAGGKVVKPDQKYAPASLP-
HYPVIR      128 QVQAGKVVSPNQSYAPANLP-
TOLCAP      127 QVEGGNVVTPDQSYAVAALG-
METRIL      131 EVANGKVVSPDKSYAPQNLP-
ROSNEC      121 ELAGGKVVSPNKSWAVTQM--
VERLON      127 QLAKAVVVKPNQSYPVQALP-
VERNON      127 QLAKAVVVKPNQNYPVQALP-
COLORB      126 QLVNGKAVSPDQSYAPQNLP-
ACRCHR      132 QLQNGKVVKPNQSYPVQALP-
COLFRU      125 QLVNGQTVNPDQSYAPQNLP-
MAGORY      127 QLVNGKAVSPDQSYAPQTLG-
MAGPOA      128 QLANGQVVKPDLSFQPQSISW
CERZEI      128 QLVNGATVQPDQSYTPQNLP-
BEABAU      122 EIAAGN-VPDKPFHVEAPKY

consensus   141 ql  g vV Pdqsy p  lp
```

Figure S2. Amino Acid sequence alignment of *P. digitatum* Sca and putative homologous from different filamentous fungi. Cysteine residues are shadowed in red. Extremely conserved amino acids are shadowed in blue in the consensus sequence. Highly conserved amino acids are shadowed in black and conserved amino acids are shadowed in gray. Abbreviations: PENDIG, *Penicillium digitatum*; PENRUB: *Penicillium rubens*; FUSOXY, *Fusarium oxysporum*; GIBFUJ, *Gibberella fujikuroi*; FUSKUR, *Fusarium kuroshium*; TRIHAR, *Trichoderma harzianum*; ASPCAL, *Aspergillus calidoustus*; EMENID, *Emericella nidulans*; TALISL, *Talaromyces islandicus*; ASPVER, *Aspergillus versicolor*; VALMAL, *Valsa malicola*; GIBMON, *Gibberella moniliformis*; TRILON, *Trichoderma longibrachiatum*; HYPJEC, *Hypocrea jecorina*; GIBZEA, *Gibberella zeae*; POCCHL, *Pochonia chlamydosporia*; PENOXA, *Penicillium oxalicum*; FUSPOA, *Fusarium poae*; HYPVIR, *Hypocrea virens*; TOLCAP, *Tolypocladium capitatum*; METRIL, *Metarhizium rileyi*; ROSNEC, *Rosellinia necatrix*; VERLON, *Verticillium longisporum*; VERNON, *Verticillium nonalfalfae*; COLORB, *Colletotrichum orbiculare*; ACRCHR, *Acremonium chrysogenum*; COLFRU, *Colletotrichum fructicola*; MAGORY, *Magnaporthe oryzae*; MAGPOA, *Magnaporthe poae*; CERZEI, *Cercospora zeina*; BEABAU, *Beauveria bassiana*.

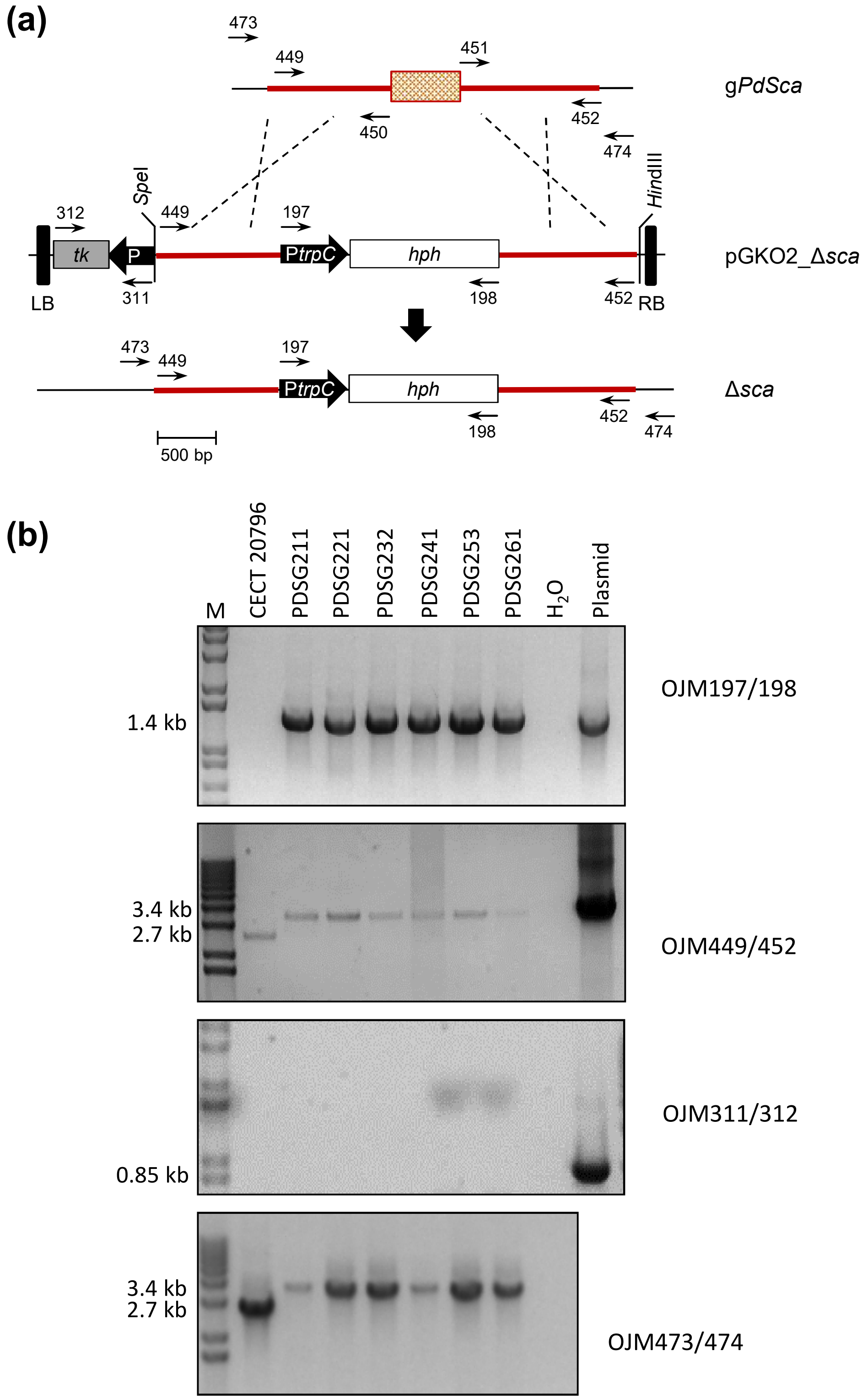

(a)
473
449
451
gPdSca
450
452
474
SpeI
HindIII
312
449
197
tk
P
PtrpC
hph
pGKO2_Δsca
LB
311
198
452
RB
473
449
197
PtrpC
hph
Δsca
198
452
474
500 bp
(b)
M
CECT 20796
PDSG211
PDSG221
PDSG232
PDSG241
PDSG253
PDSG261
H2O
Plasmid
1.4 kb
OJM197/198
3.4 kb
2.7 kb
OJM449/452
OJM311/312
0.85 kb
3.4 kb
2.7 kb
OJM473/474

Figure S3. Generation of null Δ*sca* strains. (**a**) Graphical representation of the PDIG_23520 (*sca*) gene in *P. digitatum*, the pGKO2_Δ*sca* vector designed to delete this gene and the Δ*sca* obtained. Primers use to obtain and check transformants by PCR amplification are localized in the figure. (**b**) Results of PCR amplification of genomic DNA from different *P. digitatum* strains using distinct primer pairs. In the first panel, primers OJM197/OJM198, which amplify the hygromycin marker, were used to confirm its presence in all transformants analyzed (from PDSG211 to PDSG261). The second panel displays PCR amplification using primers OJM449/OJM452 inside of the deletion construct. Different amplicon sizes were obtained between parental CECT 20796 (2.7 kb) or null transformants (3.4 kb) indicating replacement of gene by positive hygromycin marker. The third panel shows the presence of a positive fragment of 850 pb, using specific primers (OJM311/OJM312) for HSV*tk* gene, only in the pGKO2_Δ*sca*, as expected. The fourth panel supports the result obtained with the second panel, using primers (OJM473/OJM474) located outside of deletion construct.

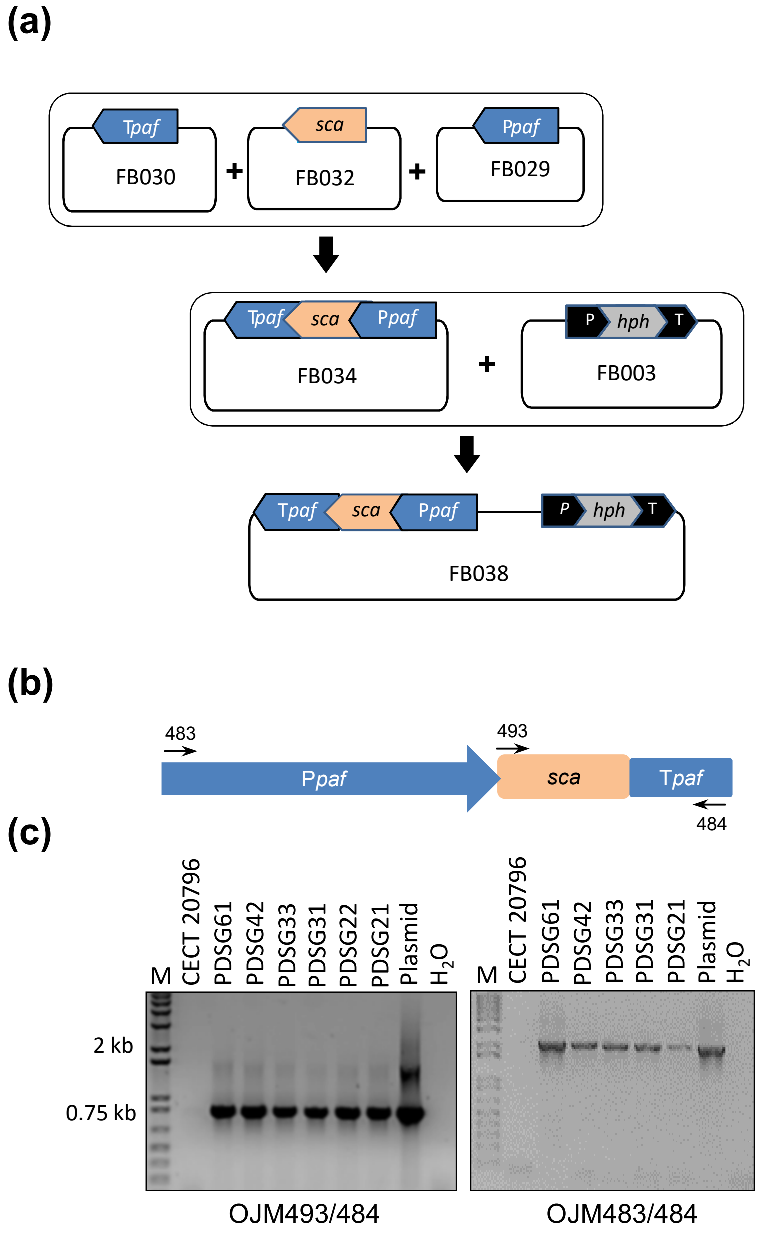

Figure S4. Generation of overproducer strains (Sca^{op}) using the FungalBraid approach. (**a**) Schematic diagram of multipartite assembly from the modular parts FB029, FB032 and FB030, to obtain transcriptional unit to produce Sca protein via use of the *paf* promoter and terminator (FB034), and binary assembly (FB034 + FB003) to obtain the final binary vector used for transformation (FB038). (**b**) Diagram to localize different primers used to confirm Sca^{op} transformants. (**c**) Results of PCR amplification of genomic DNA from different *P. digitatum* strains using several primer combinations. The left panel shows a 750 pb amplicon which corresponds to *sca* coding sequence and *paf* terminator present only in Sca^{op} (from PDSG21 to PDSG61). The right panel shows a 2 kb amplicon which corresponds to complete construction to produce Sca protein under *paf* promoter and terminator control. Note the absence of these amplicons in parental strain CECT 20796.

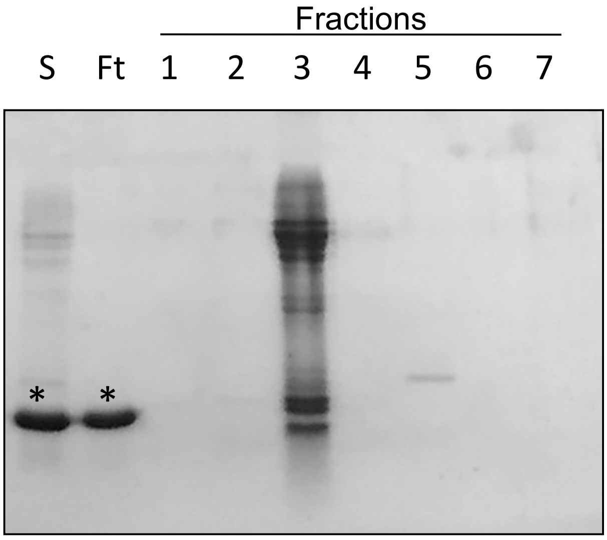

Figure S5. SDS-PAGE analysis of fractions obtained after anion exchange chromatography. Protein Sca is marked by (*). S: initial sample 10X. Ft: flow-through. Note that Sca was not adsorbed in the resin despite its predicted chemical properties (pI=4.54), and was eluted in the flow-through.

Supplementary Tables

Journal of Fungi

A novel Secreted Cysteine-rich Anionic (Sca) protein from the citrus postharvest pathogen Penicillium digitatum enhances virulence and modulates the activity of the Antifungal Protein B (AfpB)

Sandra Garrigues †, Jose F. Marcos, Paloma Manzanares, and Mónica Gandía *

Table S1. qRT-PCR primers used in this work.

Primer	Organism	Gene	Sequence 5´- 3´	Use	T_{an} (ºC)	Reference
OJM453	*P. digitatum*	PDIG_23520	CACCGTCACTTCTGGCTG	Forward	58	This work
OJM463	*P. digitatum*	PDIG_23520	GCTTGACAACCGCACCGC	Reverse	58	This work
OJM466	*P. digitatum*	PDIG_68840	GACGATCTTGATGCCCGAG	Forward	60	[22]
OJM467	*P. digitatum*	PDIG_68840	AGTCAACCCTCCTGTGGTG	Reverse	60	[22]
OJM85	*P. expansum*	*β-tubulin*	AGCGGTGACAAGTACGTTCC	Forward	65	[42]
OJM86	*P. expansum*	*β-tubulin*	ACCCTTGGCCCAGTTGTTAC	Reverse	65	[42]
OJM151	*P. digitatum*	*L18a*	TGGGGCAGAGGGAACTTGAG	Forward	65	[43]
OJM152	*P. digitatum*	*L18a*	ACCGACGCTGTTGAGGCTCT	Reverse	65	[43]
OJM334	*P. chrysogenum*	*18S rRNA*	CGACTTCAGGAAGGGGTGTA	Forward	56	[44]
OJM335	*P. chrysogenum*	*18S rRNA*	CTTGGATGTGGTAGCCGTT	Reverse	56	[44]

Table S2. PCR primers used to generate different constructs and to verify the transformants of *sca* gene.

Primer	Location	Gene	Sequence 5´- 3´	Use	Strain[a]	Reference
OJM449	5´	*sca*	CGACTAGTGTCTTACTTCTTCATTTGACCG	Forward	KO/wt/EC	This work
OJM450	5´	*sca*	ATGCTCCTTCAATATCAGTTAACGGGCTCATTGCA GGTGATATTC	Reverse	wt/EC	This work
OJM451	3´	*sca*	CCGACCGGGAACCAGTTAACATTGAGTATGAGCTG AATCCTG	Reverse	KO/wt/EC	This work
OJM452	3´	*sca*	GGAAGCTTCACCAAGTGCACAGTAGAG	Forward	wt/EC	This work
OJM473	Extern	*sca*	ATGCAATGAATCGAATGACATC	Forward	KO/wt/EC	This work
OJM474	Extern	*sca*	CCAAGGTGGATGGCTGAGC	Reverse	KO/wt/EC	This work
OJM483	Promoter	*paf*	ATCCCGGGGAATTCAGAGAGCTTTTCGTACG	Forward	Op	[47]
OJM484	Terminator	*paf*	ATTCTAGAGCAGCAGTTTGATAGTTATCCCT	Reverse	Op	[47]
OJM493	5´	*sca*	TGAGCAGACATCACCATGATGCAGCTCGCTCAGCT CATTC	Forward	Op/wt	This work
OJM197	5´	*hph*	CGTTAACTGATATTGAAGGAGCAT	Forward	KO/EC	[41]
OJM198	3´	*hph*	TGTTAACTGGTTCCCGGTCGG	Reverse	KO/EC	[41]
OJM311	5´	HSV*tk*	CCACGGAAGTCCGCCCGGAGC	Forward	EC	[41]
OJM312	3´	HSV*tk*	GACGTGCATGGAACGGAGGCG	Reverse	EC	[41]

[a]KO: knock out; wt: wild type; EC: ectopic; Op: overproducer

Table S3. Minimum inhibitory concentration (MIC) values (µg/mL) of Sca, AfpB and PeAfpA against the microorganisms tested.

Strain	Sca	AfpB	PeAfpA
Penicillium digitatum CECT 20796	>256	4	1
Penicillium italicum CECT 2294	>20	2	2
Penicillium expansum CECT 20906	>20	4	2
Penicillium chrysogenum Q176	>20	8	2
Botrytis cinerea CECT 2100	>20	12.5	4
Aspergillus niger CBS 120.49	>20	4	2
Fusarium oxysporum 4287	>200	100	4
Magnaporthe oryzae PR9	>200	>200	16
Saccharomyces cerevisiae BY4741	>50	>200	4
Escherichia coli JM109	>128	>200	>128
Bacillus subtilis CECT 498	>128	nt[a]	64

nt[a]: not tested
